# Supplementary material for: PrEP Use Awareness and Interest Cascade among MSM and Transgender Women Living in Bali, Indonesia
Source: Trop Med Infect Dis. 2020 Oct 10;5(4):158. doi: 10.3390/tropicalmed5040158 (PMC7709693; doi:10.3390/tropicalmed5040158)
Supplement: Supplementary file 1 [file tropicalmed-05-00158-s001.pdf]

Supplementary Materials: The following are available online at www.mdpi.com/2414-6366/5/4/158/s1,

Table S1. PrEP Cascade 1 (Including PrEP Awareness).

Step	Variable	N	% of total	% of previous step
1	Total Participant	220	100.0%	0.0%
	High HIV risk (CLAI, STI, > 1 sex partner)	170	77.3%	77.3%
2	Self-assessed high risks for HIV infection	129	58.6%	75.9%
3	Aware of PrEP	17	7.7%	13.2%
3	Interested in PrEP use	11	5.0%	64.7%
4	Willing to do PrEP procedures	10	4.5%	90.0%
5	Willing to pay IDR 500K–600K for PrEP	7	3.2%	70.0%
6	Already on PrEP	2	0.9%	28.6%

Table S2. PrEP Cascade 2 (Excluding PrEP Awareness).

Step	Variable	N	% of total	% of previous step
1	Total Participant	220	100.0%	0.0%
	High HIV risk (CLAI, STI, > 1 sex partner)	170	77.3%	77.3%
2	Self-assessed high risks for HIV infection	129	58.6%	75.9%
3	Interested in PrEP use	105	47.7%	81.4%
4	Willing to do PrEP procedures	82	37.3%	78.1%
5	Willing to pay IDR 500K–600K for PrEP	40	18.2%	48.8%
6	Already on PrEP	2	0.9%	5.0%

Table S3. Variance Inflation Factor Among all Independent Variables.

Variable	VIF	1/VIF
Age group		
25–29 years	1.55	0.646824
30–39 years	1.59	0.629107
40 years or above	1.25	0.801459
Education		
Senior high school	2.7	0.36975
University	3.15	0.317655
Income more than Bali minimum wage	1.52	0.659633
High social engagement as MSM/waria	1.14	0.875859
Adequate HIV risk/prevention knowledge	1.07	0.932276
Sex with women in the last 6 months	1.17	0.856007
> 1 MSM/waria sex partner in the last 6 months	1.18	0.849079
Inconsistent condom use in the last 6 months	1.15	0.869277
Paid sex in the last 6 months	1.13	0.884867
STI diagnoses in the last 6 months	1.14	0.874268
Mean VIF	1.52	
